# Response to Dr. Bernstein

**DOI:** 10.1097/PR9.0000000000000814

**Published:** 2020-03-25

**Authors:** Murat Aydede

**Affiliations:** Department of Philosophy, University of British Columbia, Vancouver, BC, Canada.

Dr. Bernstein seems to claim that my proposed reformulation of the IASP definition of pain is ambiguous. “Ambiguous” means, roughly, “having more than one interpretation or meaning.” I don't see how my proposal is ambiguous in this sense. Counterexamples and exceptions are important—a successful taxonomic definition should ideally be free of them. The examples Dr. Bernstein gives as counterexamples are not actually counterexamples that pose difficulties for my proposed restatement of the current IASP definition. Finally, Dr. Bernstein's own proposal fails to be a taxonomic definition postulating only a correspondence as it does between pains and certain kinds of experiences—not to mention other problems with it.

## Disclosures

The author has no conflicts of interest to declare.

